# Effects of Daily Zinc, Daily Multiple Micronutrient Powder, or Therapeutic Zinc Supplementation for Diarrhea Prevention on Physical Growth, Anemia, and Micronutrient Status in Rural Laotian Children: A Randomized Controlled Trial

**DOI:** 10.1016/j.jpeds.2018.11.022

**Published:** 2019-04

**Authors:** Maxwell A. Barffour, Guy-Marino Hinnouho, Sengchanh Kounnavong, K. Ryan Wessells, Kethmany Ratsavong, Bangone Bounheuang, Bigphone Chanhthavong, Dalaphone Sitthideth, Khanpaseuth Sengnam, Charles D. Arnold, Kenneth H. Brown, Sonja Y. Hess

**Affiliations:** 1 Program in International and Community Nutrition, Department of Nutrition, University of California, Davis, CA; 2 Public Health Program, College of Health and Human Services, Missouri State University, Springfield, MO; 3 Lao Tropical and Public Health Institute (Lao TPHI), Vientiane, Laos; 4 Nutrition and Global Development, Bill & Melinda Gates Foundation, Seattle, WA

**Keywords:** AGP, Alpha-1-glycoacid protein, CRP, C-reactive protein, LAZ, Length and length-for-age z scores, MUAC, Mid-upper arm circumference, ORS, Oral rehydration salts, RBP, Retinol binding protein, sTfR, Soluble transferrin receptor, WAZ, Weight and weight-for-age z scores, WHO, World Health Organization, WLZ, Weight-for-length z scores

## Abstract

**Objectives:**

To evaluate the optimal zinc supplementation strategy for improving growth and hematologic and micronutrient status in young Laotian children.

**Study design:**

In total, 3407 children aged 6-23 months were randomized to receive either daily preventive zinc tablets (7 mg/d), high-zinc, low-iron micronutrient powder (10 mg/d zinc, 6 mg/d iron, and 13 other micronutrients), therapeutic zinc supplementation for diarrhea (20 mg/d for 10 days per episode), or daily placebo powder; all were followed for ~9 months. Anthropometry, hemoglobin, zinc, and iron status were assessed at baseline and endline. Analyses were by intention-to-treat, using linear and modified Poisson regression.

**Results:**

At baseline, mean (±SD) age was 14.2 ± 5.1 months and stunting and anemia prevalence were 37.9% and 55.6%, respectively. At endline, zinc deficiency in the preventive zinc (50.7%) and micronutrient powder (59.1%) groups were significantly lower than in the therapeutic zinc (79.2%) and control groups (78.6%; *P* < .001), with no impact on stunting (37.1%-41.3% across the groups, *P* = .37). The micronutrient powder reduced iron deficiency by 44%-55% compared with other groups (*P* < .001), with no overall impact on anemia (*P* = .14). Micronutrient powder tended to reduce anemia by 11%-16% among children who were anemic at baseline (*P* = .06).

**Conclusions:**

Despite improving zinc status, preventive zinc and micronutrient powder had no impact on growth. The micronutrient powder improved iron status and tended to reduce anemia among the subset of previously anemic children.

**Trial registration:**

ClinicalTrials.govNCT02428647.

Growth faltering and anemia are common in low- and middle-income countries, likely due in part to the coexistence of multiple micronutrient deficiencies, including zinc and iron deficiencies.^1^ Zinc is involved in DNA and RNA metabolism^2^ and hence modulates cell replication, differentiation, and growth. As a result, zinc deficiency is associated with a wide spectrum of adverse health events, including linear growth restriction.^1^ Several meta-analyses have concluded that preventive zinc supplementation has a small, positive impact on linear growth.^3^, ^4^, ^5^, ^6^ A common finding of these meta-analyses was the presence of a high degree of heterogeneity across the studies, possibly related to setting, age, and other baseline characteristics of study participants. In addition, the evidence suggests that the effects of zinc on growth outcomes differed by whether zinc was delivered alone or together with other micronutrients, particularly iron.^4^, ^5^ Thus, additional information is needed regarding the specific contexts in which preventive zinc supplementation is most beneficial.

Often, zinc deficiency does not occur in isolation. Supplementation with a multiple micronutrient powder offers a low-cost option to prevent multiple micronutrient deficiencies simultaneously. The World Health Organization (WHO) and United Nations Children's Fund currently promote a micronutrient powder formulation containing 12.5 mg of iron, 4.1-5 mg of zinc, and 13 other micronutrients.^7^ Despite the proven efficacy of micronutrient powder to prevent iron deficiency and anemia,^8^ the available evidence suggests a lack of effect on growth outcomes.^8^, ^9^ In addition, there are unresolved safety concerns regarding a daily iron dose of 12.5 mg.^10^, ^11^ To address these concerns, we used a new micronutrient powder formulation in the present study that contained a lower amount of iron (6 mg/d) and a greater amount of zinc (10 mg/d) than current formulations, along with standard quantities of the 13 other micronutrients.

Therapeutic zinc supplementation (20 mg per day for 10-14 days) during episodes of diarrhea is recommended by the WHO and the United Nations Children's Fund along with oral rehydration salts (ORS) to reduce disease severity.^12^ In some settings, therapeutic zinc supplementation for diarrhea has been associated with a reduced incidence of diarrhea and enhanced growth during the 2-3 months after treatment initiation.^13^ However, evidence from other studies indicates that these benefits may accrue only during the period of treatment.^14^ Furthermore, because this strategy requires appropriate recognition of diarrhea and motivation to seek treatment, as well as access to a healthcare facility or pharmacy to obtain the supplements, coverage of therapeutic zinc supplementation programs is often low, typically reaching <30% of children in need.^15^, ^16^ Moreover, children have access to therapeutic zinc supplementation only after an episode of diarrhea occurs, so zinc prevention of the first episode would not be possible. Thus, the long-term health benefit of therapeutic zinc supplementation programs is uncertain, and more evidence is needed to compare the relative benefits of preventive vs therapeutic zinc supplementation.

The goal of the present study was to compare the effects of preventive zinc supplementation, provided either as a single micronutrient or as part of new micronutrient powder formulation containing more zinc (10 mg) and less iron (6 mg) than current formulations and therapeutic zinc supplementation, on physical growth, anemia, and biomarkers of zinc and iron status in rural Laotian children.

## Methods

### Ethical Approval

This trial and the consent procedure were approved by the National Ethics Committee for Health Research, Ministry of Health, Lao PDR, and the institutional review board of the University of California, Davis, California. The trial is registered as the Lao Zinc Study, NCT02428647 (https://clinicaltrials.gov).

### Study Design and Participants

In this double-blind, placebo controlled trial, children 6-23 months at enrollment were randomized individually to 1 of 4 interventions and followed for ~36 weeks to assess responses in physical growth, anemia, and micronutrient status.^17^ The study was implemented from September 2015 through April 2017 in rural communities in Khammouane Province, central Lao PDR. The province was selected because of a high prevalence of stunting among children <5 years,^18^ the likely high prevalence of zinc deficiency, and the lack of current programs designed to reduce the risk of micronutrient deficiencies or treat diarrhea with therapeutic zinc supplements. A 2015 pilot survey (n = 111) conducted in the province found that >60% of children 6-23 months of age were zinc deficient, based on plasma zinc concentrations <65 µg/dL.

The study area (~5300 km^2^) included 300 rural villages from 5 districts (Nongbok, Xebangfai, Mahaxay, Xaibuathong, and Yommalat). All villages in these districts were invited for enrollment, except in Nongbok. Because the prevalence of stunting in Nongbok was lower than in the other districts, only villages belonging to the catchment area of health centers with a mean stunting prevalence ≥25% were invited to participate.

### Sample Size Considerations

To detect an effect size of 0.2 in the comparison of length and weight between any 2 groups, with 90% power and 5% type 1 error rate, a total sample size of 710 children per group was required. An effect size of 0.2 corresponds to a length change of ~1.0 cm, and a weight change of ~0.26 kg, and this effect size was based on the results of previous meta-analyses, which reported mean effect sizes for linear growth and weight gain ranging from 0.12 to 0.35.^5^ Allowing for 15% attrition, a total sample of 835 children (rounded up to 850) per group was determined, and a total of 3400 children were targeted for enrollment. To detect an effect size of 0.5 in the comparison of biochemical indicators (plasma zinc, ferritin, soluble transferrin receptor [sTfR], retinol binding protein [RBP], C-reactive protein [CRP] and alpha-1-glycoacid protein [AGP], and hemoglobin) between any 2 of the 4 groups, with 90% power and 5% type 1 error rate, a total sample size of 560 children, 115 (or 140 assuming 20% attrition) per group, was required. An effect size of 0.5 for the biochemical indicators corresponds to a difference of 0.5 g/L in hemoglobin, for example. The effect size of 0.5 was based on the results of a recent meta-analysis,^5^ which reported an overall effect size of 0.6 (CI 0.44-0.77), regarding the effect of zinc supplementation on serum zinc concentrations. To ensure that we would have 140 children per group with baseline and endline biomarker data, blood collection was attempted for the first 760 children enrolled, assuming a combined attrition rate, from losses to follow-up and blood draw failures, of ~30%.

### Inclusion and Exclusion Criteria

Children were considered eligible to participate in the study if they were 6-23 months of age at enrollment, their families intended to stay in the study area for the duration of the study, were willing to accept home visits, and at least 1 of the caregivers (mother, father, legal guardian) provided written informed consent. Children were excluded from participation if they demonstrated any of the following health conditions: severe anemia (hemoglobin <70 g/L), severe wasting (defined as weight-for-height z score <–3 with respect to WHO 2006 standards),^19^ bipedal edema, severe illness warranting hospital referral, congenital abnormalities that may interfere with growth, chronic medical conditions requiring frequent medical attention, known HIV infection of the index child or the child's mother, ongoing use of micronutrient supplements, or current participation in another research study.

### Randomization

A statistician at University of California Davis randomly assigned the study ID numbers to the 4 study arms, using a block randomization scheme with block lengths of 4 or 8. In the event that multiple siblings in the target age range resided in the same household, only the youngest was enrolled. In the case of twins, both twins were assigned to the same group and received all study-related interventions and follow-up, but only one was selected randomly for inclusion in the data analyses.

### Study Interventions and Follow-Up

Children were individually randomized into 1 of 4 groups (Table I; available at www.jpeds.com): (1) the preventive zinc supplementation group, who received a daily preventive zinc supplement tablet containing 7 mg of zinc and placebo therapeutic tablets for diarrhea; (2) the micronutrient powder group, who received a daily preventive micronutrient powder containing 10 mg of zinc, 6 mg of iron and 13 other micronutrients and placebo therapeutic tablets for diarrhea; (3) the therapeutic zinc supplementation group, who received a daily placebo preventive supplement tablet and therapeutic zinc tablets containing 20 mg for 10 days for diarrhea treatment; or (4) the placebo control group, who received daily placebo preventive powder and placebo therapeutic tablets for diarrhea.

The preventive and therapeutic zinc and placebo tablets were produced by Nutriset SAS (Malaunay, France). The preventive tablets were distributed in blister packages of 8 tablets and the therapeutic tablets in blister packages containing 10 tablets per strip. The micronutrient powder and placebo powder sachets were produced by DSM Fortitech Asia Pacific (Banting, Malaysia). Standard quality control procedures were used by the manufactures to confirm the nutrient concentrations. The supplements were prelabeled (by the manufacturer) with 4 different numerical codes. In the field, the 4 numerical codes were assigned specific colors (one color per intervention group) to ensure correct delivery to children in the respective study groups. Thus, each color represented one type of preventive intervention (to be taken daily) and the corresponding therapeutic tablets for diarrhea management. In addition, caretakers for each child, irrespective of the study group, were given low-osmolarity ORS sachets for diarrhea management. Supplements and ORS were replenished during weekly home visits, as needed. Caregivers were instructed to begin diarrhea treatment with the therapeutic zinc (or placebo) tablets whenever a child had 3 or more liquid stools per day and to continue the treatment for 10 days, until the blister pack was empty. In addition, caregivers also were instructed to give ORS on any day that the child had 3 or more liquid stools per day.

At enrollment, caregivers were instructed on how to administer the study products to their child, and field workers repeated these instructions every month during one of the weekly home visits. For both the preventive and therapeutic dispersible zinc and placebo tablets, caregivers were instructed to dissolve 1 tablet with clean water or breastmilk and feed the resulting suspension to the child at least 30 minutes before or after a meal. Caregivers were instructed to mix the entire contents of a micronutrient powder or placebo powder sachet into a small amount of semisolid food that the child could easily consume. Each child was visited weekly for 36 weeks (to replenish supplements and to assess health outcomes), unless lost to follow-up. During the weekly visits, caregivers were interviewed regarding consumption of the intervention products. In addition, used and unused blister packages and sachets were collected to assess adherence.

### Study Procedures

#### Pre-Enrollment Orientation and Screening

On the day of enrollment, caregivers attended an information session, first in groups and subsequently on individual basis, during which details of the trial, including the overall goals of the study, the study interventions, duration, voluntary participation, and inclusion and exclusion criteria, were explained. After the information session, caregivers who showed interest to participate in the study were asked to sign (or fingerprint) the consent statement in the presence of an independent witness. Children with written, informed parental consent were subsequently screened for eligibility.

### Data Collection

All data were recorded electronically via a customized CommCare-HQ (Dimagi, Boston, Massachusetts) application deployed on portable Samsung tablets (Samsung Galaxy, Tab 4; Samsung Group, Seoul, South Korea). At baseline, duplicate anthropometric assessments were recorded by trained anthropometric teams, using standardized procedures. Measurements included weight to the nearest 0.02 kg (383 balance; SECA, Hamburg, Germany), recumbent length to the nearest 0.1 cm (416 length board; SECA), and mid-upper arm circumference (MUAC) to the nearest 0.1 cm (Shorr-Tape Measuring Tape; Weigh and Measure, Olney, Maryland). In the event that the duplicate measurements differed by >0.1 kg for weight, or by >0.5 cm for recumbent length and MUAC, a third independent measurement was taken.

Means were computed using the 2 measures with the lowest absolute differences. Children who were severely wasted (WLZ <–3) at baseline were excluded from participation and referred to the nearest health center or hospital. The anthropometric assessments were repeated after 18 weeks and at endline (32-40 weeks). At baseline, maternal weights were measured to 0.05-kg precision (SECA 874) and maternal heights to 0.1 cm precision (SECA 213). Anthropometric teams were systematically standardized.^20^ During a total of 4 standardization sessions, the mean technical error of measurement and coefficient of reliability for length were 0.38 cm and 97%, respectively.^20^

Anemia status, based on capillary hemoglobin concentrations, was assessed for all children at baseline and endline using a Hemocue Hb 301 System (Hemocue AB, Angelholm, Sweden). The Hemocue devices was checked weekly against a commercial quality control sample (Eurotrol, Inc, Elizabethtown, Kentucky). For the purpose of evaluating biomarkers of nutritional status and health, ~7 mL samples of venous blood were collected by trained nurses from a subsample of 760 children, using evacuated, trace element-free, 7.5 mL of lithium–heparin tubes (Sarstedt AG & Co, Numbrecht, Germany). The heparinized samples were maintained at 4-8°C in portable cooler boxes until transported to field laboratories for plasma processing within ~3 hours. In the field laboratory, the blood samples were centrifuged (PowerSpin Centrifuge Model LX C856; United Products & Instruments, Inc, Dayton, New Jersey) at 1097*g* (3100 rpm) for 10 minutes and the plasma was aliquoted into clear or amber microcentrifuge tubes (0.2-1.5 mL per tube), depending on the preplanned analyses. Plasma samples intended for nutritional biomarker assays were stored in the field laboratories at –20°C and later shipped on dry ice to a permanent laboratory at the University of California, Davis, California, where the samples were transferred to another –20°C freezer.

### Laboratory Analyses

Plasma zinc was analyzed by inductively coupled plasma optical emission spectrophotometry (5100 ICP-OES SVDV, Agilent, Santa Clara, California) at the Children's Hospital of Oakland Research Institute (Oakland, California). Details of the plasma zinc assay by inductively coupled plasma optical emission spectrophotometry have been described previously.^21^ To summarize, after overnight digestion in trace element grade 70% nitric acid at 60°C, the plasma–nitric acid mixture was diluted to a final concentration of 5.5% nitric acid and then centrifuged for 10 minutes at 3000*g*. Plasma samples were analyzed in duplicate in the same run. In the event that concentrations of the duplicates differed by 10%, the analyses were repeated. Each batch of samples was analyzed along with a reference sample (Seronorm Trace Element Serum L-1 and L-2; Accurate Chemical and Scientific Corp, Westbury, New York). Iron status markers (ferritin and sTfR), RBP, and inflammatory markers (CRP and AGP) were measured using a sandwiched enzyme-linked immunosorbent assay technique at the VitMin Lab (Willstaett, Germany).^22^

### Data Entry and Analyses

A statistical analysis plan was developed and published before analyses^23^ and was strictly followed to minimize bias. The groups' identities were revealed only after the data analyses were completed and the study investigators reached consensus on the interpretation of results. All analyses were performed with STATA statistical software, release 13 (StataCorp, Austin, Texas) and SAS version 9.4 (SAS Institute, Cary, North Carolina).

### Definitions

Both absolute and standardized anthropometric measures were used in defining growth outcomes,^24^ and z scores were calculated based on the WHO Child Growth Standards. We evaluated intervention effects on the following set of primary outcomes: (1) length and length-for-age z scores (LAZ); (2) weight and weight-for-age z-scores (WAZ); (3) weight-for-length z scores (WLZ); (4) MUAC, (5) stunting (LAZ <–2 z scores), underweight (WAZ <–2 z scores), and wasting (WLZ <–2 z-scores); and (6) mean hemoglobin and anemia (hemoglobin <110 g/L). Zinc deficiency was defined as plasma zinc <65 µg/dL, storage iron deficiency as low ferritin (<12 µg/L), and iron deficiency anemia as anemia with low ferritin.^25^ Elevated sTfR, indicative of functional iron deficiency, was defined as sTfR>8.3 mg/L.^25^ Because the RBP assays produced concentrations ~13% greater than the NIST standards, vitamin A deficiency was defined as RBP <0.81 µmol/L instead of the commonly used cut-off of 0.7 µmol/L.^26^ Infant and young children feeding practices were assessed based on caregiver recall using structured survey questions as suggested by WHO.^27^ Food security was assessed using the Household Food Insecurity Access Scale.^28^ Indices of socioeconomic status, hygiene and sanitation, and water quality were developed using principal component analyses of available baseline household-level indicators, including land ownership, number of livestock and motorized vehicles, source of drinking water, availability and type of toilet, and income and education level of household head, among others.^29^

### Statistical Analyses

All analyses were done on an intention-to-treat basis among children with available data.^30^ In all analyses, the intervention group was considered the primary exposure variable. Models first assessed a global difference in treatment effect using a likelihood ratio test, and post-hoc pairwise differences were assessed subsequently in the event of a statistically significant global difference (global *P* value < .05). In all cases of statistically significant pair-wise comparisons, multiple hypothesis testing adjustments were made to determine the sensitivity of estimates.

For anthropometric and anemia outcomes, ANCOVA or modified Poisson regression methods were used to model continuous and binary outcomes, respectively. For each outcome, treatment effect parameters were estimated using minimally adjusted models that controlled for baseline measurement of the respective outcome, age at enrollment, sex, and district as the only covariates. In the assessment of treatment effects on the nutritional biomarkers (both continuous and dichotomous), separate models were constructed using either measured concentrations, or the inflammation-adjusted concentration calculated by adapting procedures recommended by the Biomarkers Reflecting Inflammation and Nutritional Determinant of Anemia project.^31^, ^32^, ^33^ Specifically, adjustment factors reflecting the changes in nutritional biomarkers during inflammation were determined by pooling together the samples collected at baseline from all 4 groups. The adjustment factors (ie, regression coefficient for CRP and/or AGP) were subsequently applied to both baseline and endline samples. In adapting the approach of the Biomarkers Reflecting Inflammation and Nutritional Determinant of Anemia project, we used the 10th percentile of the CRP and AGP concentrations in this population. In addition, adjustment factors for CRP and/or AGP were applied only if the biomarker was significantly associated with CRP or AGP (*P* < .05 for the regression coefficient). In all models, ferritin, zinc, sTfR, and RBP were log-transformed. Potential effect modification by baseline variables was explored by incorporating interaction terms in the statistical models and further investigated if marginally significant (*P* < .1). The full list of the specific covariates and effect modifiers is available in the published analysis plan.^23^

## Results

**Figure 1:**
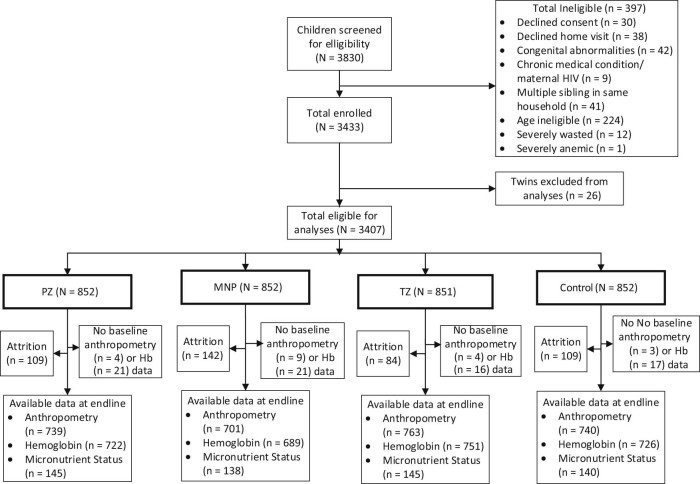
Consort diagram of study participant progression through the growth and biochemical assessments in the Lao Zinc Study. The micronutrient powder group received 1 micronutrient powder sachet daily (vitamin A [400 µg of retinol equivalents], thiamin [0.5 mg], riboflavin [0.5 mg], niacin [6 mg], vitamin B6 [0.5 mg], folic acid [150 µg of dietary folate equivalents], cyanocobalamin [0.9 µg], ascorbic acid [30 mg], cholecalciferol [5 mg], dl-α-tocopheryl acetate [5 mg of tocopherol equivalents], copper sulfate anhydrous [to provide 0.56 mg copper], potassium iodate [to provide 90 µg iodine], ferrous fumarate [to provide 6 mg of iron], selenium selenite [to provide 17 µg of selenium], zinc gluconate [to provide 10 mg of zinc]) and placebo tablet (1 per day for 10 days) during diarrhea episodes. The preventive zinc group (preventive zinc supplements) received 1 zinc tablet (7 mg/d) daily and placebo tablet (1 per day for 10 days) during diarrhea episodes. The therapeutic zinc group (therapeutic zinc supplements) received 1 placebo tablet daily and a 20-mg zinc tablet (1 per day for 10 days) during diarrhea episodes. Controls received 1 placebo powder sachet daily and placebo tablet (1 per day for 10 days) during diarrhea episodes. All children received ORS during diarrhea episodes, regardless of group allocation.

Of the 3830 children screened, 3433 were eligible for enrollment (Figure 1; available at www.jpeds.com). This included 3407 individually randomized children and 26 children from a twin pair (who were excluded at the analytic stage to ensure that only one child per household remained in the data set for analysis). Attrition during the 36 weeks of follow-up was 10% in the therapeutic zinc group, 13% in preventive zinc and control groups, and 17% in the micronutrient powder group (*P* = .01). The children lost to follow-up were statistically similar to those who completed the study with respect to baseline age, maternal variables, and anemia (Table II; available at www.jpeds.com). Similarly, baseline anthropometric indicators were comparable between those who completed the study vs those who dropped out, except for MUAC, which was slightly, but significantly, lower (*P* = .04) in the children who completed the study (13.8 ± 1.0 cm) compared with those lost to follow-up (13.9 ± 1.1 cm).

The average age at baseline was 14.3 ± 5.0 months (Table III). The prevalences of stunting and anemia were 40% and 55%, respectively. Approximately 73% of children were breastfed in the previous 4 weeks, and only 14% met the WHO definition of adequate dietary diversity. Overall reported adherence to the preventive supplement was 91%, ranging from 89% in the micronutrient powder group to 92% in the preventive zinc and therapeutic zinc groups (*P* < .01; Table IV [available at www.jpeds.com]). This amounted to a daily supplemental zinc intake of ~6.5 mg for the preventive zinc and ~9.0 mg for the micronutrient powder group over the duration of follow-up. Children received diarrhea treatment for an average of 4 days per 100 child days. Children in the therapeutic zinc group received the equivalent of 0.8 mg/d zinc over the course of the study (Table IV).

**Table III:** Group-wise comparison of baseline characteristics in children included in the analyses

Characteristics	All (n = 2943)	Preventive zinc (n = 738)	Micronutrient powder (n = 701)	Therapeutic zinc (n = 764)	Control (n = 740)
Child characteristics*					
Age, mo	14.3 ± 5.0	14.1 ± 5.1	14.3 ± 5.0	14.5 ± 5.2	14.1 ± 5.1
Male, n (%)	1503 (51.1)	370 (50.1)	356 (50.8)	392 (51.3)	385 (52.0)
Length, cm	72.5 ± 5.5	72.1 ± 5.5	72.5 ± 5.4	72.6 ± 5.7	72.5 ± 5.6
Weight, kg	8.3 ± 1.3	8.2 ± 1.3	8.3 ± 1.3	8.3 ± 1.3	8.2 ± 1.3
MUAC, cm	13.8 ± 1.0	13.7 ± 1.0	13.8 ± 1.0	13.8 ± 1.0	13.8 ± 1.0
LAZ	−1.75 ± 1.08	−1.78 ± 1.03	−1.76 ± 1.07	−1.78 ± 1.11	−1.67 ± 1.09
WAZ	−1.44 ± 1.01	−1.48 ± 1.00	−1.46 ± 1.02	−1.43 ± 1.03	−1.37 ± 1.00
WLZ	−0.71 ± 0.96	−0.74 ± 0.97	−0.73 ± 0.96	−0.69 ± 0.98	−0.67 ± 0.94
Stunting, n (%)	1167 (39.7)	303 (41.1)	288 (41.1)	300 (39.3)	276 (37.3)
Underweight, n (%)	808 (27.5)	209 (28.3)	212 (30.2)	211 (27.6)	176 (23.8)
Wasting, n (%)	243 (8.3)	72 (9.8)	58 (8.3)	58 (7.6)	55 (7.4)
Hemoglobin, g/L	107.0 ± 1.1	107.6 ± 1.1	107.5 ± 1.1	107.8 ± 1.1	108.0 ± 1.0
Anemia, n (%)	1614 (54.8)	406 (55.0)	396 (56.5)	415 (54.3)	397 (53.7)
Complementary feeding					
Breastfeeding, n (%)	2107 (72.6)	529 (73.9)	551 (76.1)	516 (70.4)	511 (70.2)
Adequate dietary diversity, n (%)	413 (14.2)	107 (14.9)	91 (12.5)	108 (14.7)	107 (14.7)
Iron-rich foods, n (%)	1948 (67.0)	478 (66.8)	478 (65.8)	484 (65.9)	508 (69.6)
Household/maternal*					
District, n (%)					
Xebangfai	740 (25.1)	183 (24.8)	184 (26.1)	190 (24.9)	184 (24.9)
Nongbok	449 (15.3)	112 (15.2)	104 (14.8)	119 (15.6)	114 (15.4)
Mahaxay	734 (25.0)	181 (24.5)	178 (25.4)	188 (24.6)	187 (25.3)
Xaibuathong	585 (19.9)	152 (20.6)	132 (18.8)	156 (20.5)	145 (19.6)
Yommalat	434 (14.7)	110 (14.9)	104 (14.8)	110 (14.4)	110 (14.9)
HFIAS	3.2 ± 5.0	3.2 ± 5.0	3.2 ± 5.0	3.2 ± 5.0	3.1 ± 4.0
Maternal age, y	26.8 ± 5.9	26.5 ± 5.7	26.8 ± 5.9	26.7 ± 6.0	27.0 ± 6.0
Maternal BMI, kg/m^2^	21.4 ± 2.9	21.4 ± 3.0	21.5 ± 2.7	21.4 ± 2.9	21.4 ± 3.0
Micronutrients (n = 568)†					
Plasma zinc, µg/dL	54.2 (14.2)	52.2 (16.6)	55.2 (13.8)	54.4 (13.0)	55.1 (13.5)
Zinc deficiency, n (%)	432 (75.4)	110 (74.3)	101 (72.1)	108 (74.5)	113 (80.7)
Ferritin, µg/L	31.0 (32.6)	30.3 (32.7)	27.7 (26.6)	27.1 (21.6)	27.2 (30.5)
Storage ID, n (%)	150 (26.1)	36 (24.3)	39 (27.9)	37 (25.3)	38 (27.1)
sTfR, mg/L	9.4 (7.2)	9.1 (7.6)	9.6 (6.0)	9.3 (6.2)	9.4 (7.6)
Functional ID, n (%)	379 (66.0)	91 (61.5)	97 (69.3)	99 (67.8)	92 (65.7)
RBP, µmol/L	1.3 (0.4)	1.1 (0.4)	1.1 (0.4)	1.3 (0.3)	1.2 (0.3)
VAD, n (%)	9 (1.6)	3 (2.0)	4 (2.7)	1 (0.7)	1 (0.7)
Inflammation (n = 568)†					
AGP, g/L	0.61 (0.42)	0.64 (0.44)	0.63 (0.47)	0.60 (0.41)	0.60 (0.41)
AGP>1 g/L, n (%)	119 (20.7)	34 (23.0)	31 (22.1)	26 (17.8)	28 (20.0)
CRP, mg/L	0.47 (1.60)	0.57 (1.55)	0.41 (1.19)	0.43 (1.47)	0.46 (2.31)
CRP >5 mg/L, n (%)	67 (11.7)	18 (12.2)	13 (9.3)	16 (11.0)	20 (14.3)

*BMI*, body mass index; *HFIAS*, Household Food Insecurity and Access Score.

Zinc deficiency defined as zinc <65 µg/dL, Storage ID as ferritin <12 µg/L, Functional ID as sTfR >8.3 mg/L, and vitamin A deficiency defined as RBP <0.81 µmol/L.

* Values represent mean ± SD for continuous variable or n (%) for dichotomized variables.

† Values represent median (IQR) for continuous variable or n (%) for dichotomized variables.

At endline, the adjusted mean length (79.1 ± 4.9 cm), mean weight (9.6 ± 1.3 kg), and mean MUAC (14.0 ± 1.0 cm) did not differ by study group (Table V). Likewise, the mean LAZ (–1.94 ± 1.0), WAZ (–1.52 ± 1.0), and WLZ (–0.72 ± 0.93) were similar across the 4 groups. These standardized anthropometric scores declined in all groups relative to their corresponding baseline values. There was no treatment effect on stunting (44%-50% across the groups; *P* = .37) or underweight (26%-30% across the groups; *P* = .45). Wasting prevalence differed significantly by study group (*P* = .02) and was significantly lower in the preventive zinc group (4.7%; 95% CI 3.7%-7.0%) compared with the therapeutic zinc group (7.6%; 95% CI 5.5%-9.2%). There were no other significant pair-wise differences in wasting prevalence. A secondary analyses of anthropometry outcomes assessed at midline (~18 months) did not find any treatment effects (data not shown).

**Table V:** Effects of 32-40 weeks of supplementation with daily preventive zinc supplements, daily micronutrient powder, or therapeutic zinc supplements for diarrhea on final absolute and standardized anthropometric indices and biochemical outcomes among rural Laotian children

Outcome	Preventive zinc	Micronutrient powder	Therapeutic zinc	Control	*P* value*
Anthropometry					
n	739	701	763	740	–
Length, cm	79.0 ± 4.8	79.2 ± 4.8	79.2 ± 5.0	79.3 ± 4.9	.41
Weight, kg	9.52 ± 1.25	9.58 ± 1.32	9.63 ± 1.41	9.63 ± 1.32	.92
MUAC, cm	14.0 ± 0.9	14.0 ± 1.0	14.0 ± 1.0	14.0 ± 0.9	.71
LAZ	−1.93 ± 0.97	−1.94 ± 1.00	−1.95 ± 1.02	−1.93 ± 1.00	.58
WAZ	−1.51 ± 0.88	−1.51 ± 0.93	−1.53 ± 0.97	−1.52 ± 0.90	.76
WLZ	−0.72 ± 0.79	−0.72 ± 0.85	−0.73 ± 0.89	−0.74 ± 0.82	.92
Stunting, n (%)	355 (47.0)	337 (47.0)	374 (49.6)	317 (44.4)	.37
Underweight, n (%)	217 (28.9)	197 (26.1)	219 (28.5)	203 (30.4)	.45
Wasting, n (%)	38 (4.7)^a^	39 (6.2)^ab^	57 (7.6)^b^	35 (4.7)^ab^	.02
Anemia					
n	722	689	751	726	—
Hemoglobin, g/L	110.4 ± 0.4	111.6 ± 0.4	110.6 ± 0.4	110.6 ± 0.4	.09
Anemia, n (%)	324 (44.9)	274 (39.2)	315 (42.4)	313 (43.2)	.14
Micronutrient status					
Inflammation, unadjusted					
n	145	138	145	140	
Plasma zinc, µg/dL	63.4 (61.0, 65.7)^c^	59.7 (57.6, 62.1)^a^	54.4 (52.4, 56.4)^b^	53.6 (51.6, 55.6)^b^	<.001
Zinc deficiency, %	87 (60.0)^a^	92 (67.1)^a^	122 (84.7)^b^	119 (84.5)^b^	<.001
Ferritin, µg/L	28.1 (25.2, 31.0)^b^	37.2 (33.3, 41.1)^a^	26.0 (23.3, 28.6)^b^	26.7 (23.8, 29.3)^b^	<.001
Storage ID (%)	20 (14.2)^b^	6 (4.8)^a^	28 (17.6)^b^	22 (15.4)^b^	.02
sTfR, mg/L	10.2 (9.8, 10.7)^b^	9.5 (9.1, 9.9)^a^	10.3 (9.9, 10.7)^b^	10.4 (10.0, 10.9)^b^	.02
Functional ID (%)	92 (63.9)	78 (58.2)	91 (62.4)	92 (65.9)	.49
RBP, µmol/L	1.19 (1.14, 1.23)	1.22 (1.18, 1.27)	1.23 (1.19, 1.28)	1.20 (1.16, 1.25)	.49
VAD	11 (7.2)	12 (9.0)	11 (8.0)	7 (4.8)	.54
Inflammation-adjusted†					
n	145	138	145	140	
Zinc, µg/dL	66.4 (64.0, 68.9)^c^	62.2 (59.8, 64.5)^a^	56.8 (54.7, 58.9)^b^	56.0 (53.9, 58.1)^b^	<.001
Zinc deficiency (%)	74 (50.7)^a^	81 (59.1)^a^	114 (79.2)^b^	111 (78.6)^b^	<.001
Ferritin, µg/L	18.9 (17.2, 20.7)^b^	26.0 (23.5, 28.5)^a^	18.0 (16.4, 19.7)^b^	18.4 (16.7, 20.1)^b^	<.001
Storage ID (%)	33 (17.0)^b^	16 (5.8)^a^	42 (21.1)^b^	40 (20.3)^b^	<.01
sTfR, mg/L	—	—	—	—	—
Functional ID (%)	—	—	—	—	—
RBP, µmol/L	1.33 (1.28, 1.37)	1.33 (1.29, 1.38)	1.35 (1.31, 1.40)	1.33 (1.29, 1.38)	.58
VAD‡	—	—	—	—	—
Inflammation					
n	145	138	145	140	
CRP, mg/L	0.68 ± 0.08	0.47 ± 0.06	0.58 ± 0.07	0.57 ± 0.08	.17
AGP, g/L	0.69 ± 0.03	0.65 ± 0.03	0.64 ± 0.03	0.65 ± 0.03	.59

*ID*, iron deficiency; *VAD*, vitamin A deficiency.

Values represent mean ± SD for continuous anthropometric values; geometric mean (95% CI) for continuous nutritional biomarkers, frequency (marginal prevalence) for all dichotomous variables and geometric ± standard error for CRP and AGP.

* Values on the same row with different superscript are significantly different (*P* < .05) after adjustment for age, sex, and district baseline status. Zinc deficiency defined as zinc < 65 µg/dL, Storage ID as ferritin <12 µg/L, Functional ID as sTfR >8.3 mg/L, and VAD defined as RBP <0.81 µmol/L.

† In adjusting for inflammation, adjustment factors were estimated by pooling together baseline data across the 4 groups and subsequently using procedures recommended by the Biomarkers Reflecting Inflammation and Nutritional Determinant of Anemia project to estimate CRP and AGP coefficients. There was no significant association between baseline sTfR and CRP or AGP. Hence, endline sTfR was not adjusted for inflammation.

‡ Prevalence too low to model treatment effects.

At endline, mean inflammation-adjusted plasma zinc concentrations in the preventive zinc (66.4 µg/dL [95% CI 64.0-68.9]) and micronutrient powder (62.2 µg/dL [95% CI 59.8-64.5]) groups were significantly greater than in the therapeutic zinc (56.8 µg/dL [95% CI 54.7-58.9]) and control (56.0 µg/dL [95% CI 53.9-58.1]) groups (Table V, *P* < .001), resulting in a significantly lower prevalence of zinc deficiency (*P* < .001) in preventive zinc (60%) and micronutrient powder (67%) groups compared with the therapeutic zinc (85%) and control (85%) groups. Ferritin concentration in the micronutrient powder group (26.0 mg/L [95% CI 23.5-28.5]) was greater (*P* < .001) than the other 3 groups (~18 mg/L, Table V), translating into ~44-55% reduction in iron deficiency. The micronutrient powder resulted in lower sTfR concentrations, with no impact on functional iron deficiency (Table V). We found no treatment effects on RBP, CRP, or AGP.

**Figure 2:**
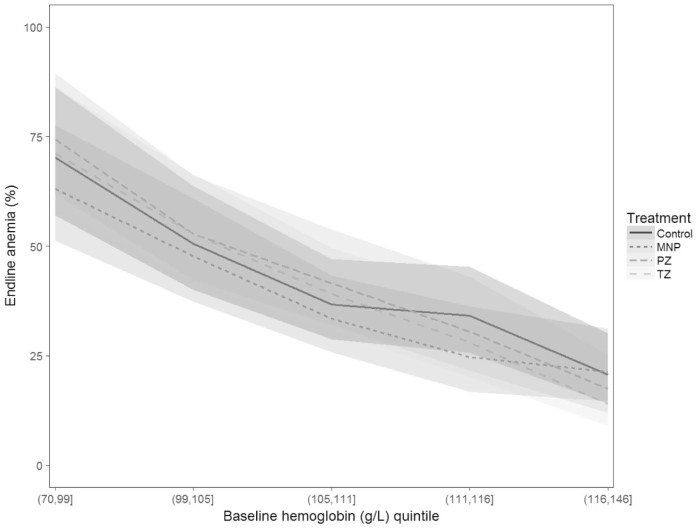
Effects of 32-40 weeks of supplementation with daily preventive zinc supplements, micronutrient powder, or therapeutic zinc supplements for diarrhea on anemia prevalence among rural Laotian children, stratified by baseline hemoglobin concentrations. *Models adjusted for age, sex, district, and baseline hemoglobin. No effects of micronutrient powder on anemia in previously nonanemic children (baseline Hb ≥110; *P* > .05 for all pairwise comparisons); in previously anemic children (baseline Hb <110), the micronutrient powder reduced the prevalence of anemia by 9 percentage points (vs preventive zinc; *P* = .008), by 7 percentage points (vs therapeutic zinc; *P* = .041) and by 6 percentage points (vs control, *P* = .08). *MNP*, micronutrient powder; *PZ*, preventive zinc; *TZ*, therapeutic zinc.

There was an overall marginal effect of the study group on hemoglobin concentration (*P* = .09) but not anemia prevalence (*P* = .14) (Table V). Specifically, the final hemoglobin concentration in the micronutrient powder group (111.6 ± 0.4 g/L) was greater compared with the preventive zinc (110.4 ± 0.4 g/L), therapeutic zinc (110.6 ± 0.4 g/L), and control (110.6 g/L ± 0.4) groups. We observed an interaction-by-baseline hemoglobin status (*P* = .06), such that among children who were anemic at baseline, the micronutrient powder was associated with a trend to a reduction in anemia of 6-9 percentage points (global *P* = .06) compared with the other 3 groups (Figure 2), whereas there was no impact among nonanemic children. The micronutrient powder did not prevent the development of anemia among previously nonanemic children (Table VI; available at www.jpeds.com). The prevalence of anemia among previously nonanemic children was 24%-28% (Table VI) and did not differ by group allocation (*P* = .62).

Complementary feeding practices did not differ over the course of the study. Based on data pooled from 9 monthly caregiver dietary recalls, the percentage of times children met the WHO recommended criteria for minimum dietary diversity (~29%, *P* = .72) and minimum meal frequency (~61%, *P* = .72), and consumption of animal source foods (~90%, *P* = .18) and iron-rich foods (~84%, *P* = .44) did not differ by group (Table VII; available at www.jpeds.com).

## Discussion

The results of this community-based, randomized controlled trial indicate that daily preventive zinc supplements providing 7 mg zinc/d and daily micronutrient powder sachets containing 10 mg zinc, despite improving plasma zinc concentrations, had no impact on linear growth or weight gain among young children residing in rural health districts of central Lao PDR. Therapeutic zinc supplementation given for the treatment of diarrhea had no effect on zinc status nor linear growth. In addition, the low-iron, high-zinc micronutrient powder resulted in a significant positive effect on iron status and an overall marginal effect on hemoglobin. Moreover, the micronutrient powder had a marginally significant positive effect on hemoglobin and anemia in children who were anemic at baseline.

The lack of impact of daily preventive zinc supplementation on physical growth in the present study differs from the results of 4 systematic reviews and meta-analyses on preventive zinc supplementation, all of which found a small positive effect on linear growth and weight gain, albeit with significant heterogeneity of responses across trials.^3^, ^4^, ^5^, ^6^ The failure of the high-zinc, low iron micronutrient powder to increase linear growth is consistent with previous meta-analyses of micronutrient powder, which did not find an effect of micronutrient powder on growth.^8^ The lack of any growth impact of therapeutic zinc is not surprising, given the low frequency of therapeutic supplementation and correspondingly small amount of supplemental zinc consumed over the course of the trial by the children in this study group. Our findings regarding therapeutic zinc and growth are consistent with previous studies, which found no impact of short-term zinc supplementation of acutely ill children on their subsequent growth^14^, ^34^ and highlights the need for additional interventions beyond the period of zinc treatment.

Possible reasons for the lack of any growth effect in the present study may be that the study participants were not sufficiently growth restricted or zinc deficient to be able to respond to supplementation, the dose or duration of supplementation was insufficient, possibly because of impaired intestinal absorption, or the children did not adhere to the supplementation protocol. As ~40% of children were stunted and ~75% had low plasma zinc concentration, the children should have been able to respond to supplemental zinc if zinc deficiency was the only factor restricting their growth. Moreover, we found no evidence of effect modification by baseline LAZ, suggesting that even the more severely growth-restricted children did not respond to zinc supplementation. However, the prevalence of chronic inflammation in this population (21% with elevated AGP concentrations) may have had constraining effects on growth. The dose and duration of preventive zinc tablet were consistent with the range of dosing regimens used in earlier studies that did find a growth response. Thus, the dosing regimen does not seem to explain the lack of observed growth impact. Some studies have shown that the absorption of zinc from micronutrient powder is low^35^ and that the efficacy of zinc may be compromised when zinc is coadministered with iron.^36^ The dose of 10 mg of zinc used in the micronutrient powder was based on consideration of both the amount of zinc absorbed when mixed with food (<50%)^35^ and the dose of zinc in micronutrient powder previously shown to increase plasma or serum zinc concentration.^37^, ^38^ The fact that both the micronutrient powder and preventive zinc significantly increased plasma zinc concentration relative to the control group provides evidence that the supplemental zinc was being consumed and absorbed, and the most plausible explanation for the lack of growth effect is that growth faltering in this study population may be driven by other factors that are not responsive to zinc supplementation. Additional research is needed to fully understand the etiology of growth faltering in this population. The statistically significant effect of preventive zinc on wasting is of small magnitude and may represent a spurious finding in view of the lack of any independent effects of the supplements on length and weight gain.

In settings with a high burden of infections, there is interest in finding the minimum effective iron dose necessary for reducing anemia, without increasing the risk for adverse health outcomes.^10^, ^39^ Proposed strategies for achieving this include daily supplementation with 10-12.5 mg for just 3 consecutive months a year and intermittent iron supplementation a few days a week instead of daily.^40^ The limited evidence on these strategies suggest an overall efficacy (compared with placebo controls) in reducing anemia, albeit with a lower effect size compared with delivering 12.5 mg iron daily.^41^, ^42^, ^43^ In the current study, we intentionally reduced the amount of iron in the micronutrient powder in an attempt to avoid possible adverse effects of iron supplementation. Our results are consistent with this pattern of lower efficacy compared with previous studies of daily supplementation with greater amounts of iron for 2-12 months.^8^, ^44^ Although the present study found a marginal impact on anemia prevalence, this effect was lower than in previous micronutrient powder trials,^8^ possibly due to the lower iron content (6 mg iron /day) in the micronutrient powder provided in the present study. Even in children who were anemic, in whom the micronutrient powder increased hemoglobin concentration and reduced the prevalence of anemia, the relative reduction in anemia prevalence was <16%, compared with ~30% found in trials delivering 12.5 mg of iron.^8^

In neighboring Savannakhet (south of the study area), an earlier study by Kounnavong et al reported that in children 6-52 months, daily or twice-weekly micronutrient powder containing 12.5 mg of iron was associated with a greater reduction in anemia (32-35 percentage points decrease) compared with children receiving no micronutrient powder (only 10 percentage points decrease) during 24 weeks of follow-up.^45^ The magnitude of effect reported by Kounnavong et al was greater than observed in our study population (6-9 percentage point reduction), despite a lower reported compliance (73%; defined as consumption on at least 5 days in a week) and a shorter duration of follow-up (up to 24 weeks) in that study.^45^ This difference in effect may suggest that a greater iron-dose micronutrient powder, even if given intermittently, may be more efficacious than the lower daily dose used in this study. Perhaps more importantly, the marginal response in anemia despite a substantial relative reduction in iron deficiency suggests the presence of other cause of anemia in this population. This view also is supported by the fact that the micronutrient powder did not prevent the incidence of new anemia cases among children who were not anemic at baseline. In Cambodian children, Wieringa et al concluded that hemoglobinopathies, and not iron deficiency, may be more relevant to the burden of anemia.^46^ Additional evidence is needed to determine the cause-specific attribution fractions for anemia with respect to iron deficiency, hemoglobinopathies, and infections,^46^, ^47^ to determine optimal anemia control strategies.

Several strengths of this study include its implementation in a zinc-deficient and thus potentially responsive population, the individual randomization protocol, the frequency of follow-up visits, and the masking procedures used to ensure continued blinding throughout the field implementation and data analyses. In addition, the drop-out rate of ~13% was lower than expected, ensuring a sufficient final sample size for addressing the proposed questions. As indicated previously, the baseline characteristics of the children lost to follow-up were comparable with those who remained in the study. Moreover, among the children lost to follow-up, there were no differences in age, weight, length, and hemoglobin between the groups at baseline.

A potential weakness of this trial is the nature of the comparison groups. The micronutrient powder and control preventive interventions were formulated in powder forms, whereas the preventive zinc and therapeutic zinc were formulated in tablet forms, potentially undermining the blinding procedure. However, because there were 2 powder groups, and 2 tablet groups, it was impossible to identify the exact intervention allocation for any particular group. A related problem is that micronutrient powder is designed to be taken along with food, whereas the preventive zinc is recommended to be consumed between meals. Thus, it is possible that the children's dietary patterns were affected differently, possibly confounding treatment effects. However, analyses of the dietary data found no difference in meal frequency, dietary diversity, consumption of iron-rich foods, and animal source foods (Table VII) across the groups over the course of the follow-up period, and adjusting for these variables did not change the estimated impact on growth and anemia outcomes.

In conclusion, our data suggest that preventive zinc supplements, provided alone or in combination with other micronutrients at doses of 7-10 mg zinc/d to young children in rural central Lao PDR, improved biomarkers of zinc status but had no impact on physical growth. Also, because the micronutrient powder tended to improve iron status and reduce anemia among initially anemic children, this may be the preferred strategy to deliver zinc and other micronutrients in this population. Additional research is needed to understand the optimal strategy for reducing the high burden of stunting and anemia in this population.

Acknowledgments available at www.jpeds.com

## Figures and Tables

**Table I:** Preventive and therapeutic supplements received by each study group of the Lao Zinc Study

Supplements	Preventive zinc	Micronutrient powder	Therapeutic zinc	Control
Preventive supplement	Zinc-containing tablet (7 mg/d)	Micronutrient powder (containing 10 mg of zinc, 6 mg of iron, + 13 other micronutrients*)	Placebo tablet	Placebo powder
Therapeutic supplement for diarrhea	ORS + placebo tablet	ORS + placebo tablet	ORS + zinc tablet (20 mg/d) for 10 d during acute diarrhea episodes	ORS + placebo tablet

* Micronutrient powder contents of 1 daily dose micronutrient powder sachet: vitamin A (400 µg Retinol Equivalents), thiamin (0.5 mg), riboflavin (0.5 mg), niacin (6 mg), vitamin B6 (0.5 mg), folic acid (150 µg of Dietry Folate Equivalents), cyanocobalamin (0.9 µg), ascorbic acid (30 mg), cholecalciferol (5 mg), dl-α-tocopheryl acetate (5 mg of Tocopherol Equivalents), copper sulfate anhydrous (0.56 mg of copper), potassium iodate (90 µg of iodine), ferrous fumarate (6 mg of iron), selenium selenite (17 µg of selenium), and zinc gluconate (10 mg of zinc).

**Table IV:** Caregiver report of adherence to study interventions among children participating in the Lao Zinc Study

Adherence types*	Preventive zinc	Micronutrient powder	Therapeutic zinc	Control	*P* value
Observation days	194 603	186 133	198 975	195 804	
Days preventive tablet/sachet given, n (%)	179 299 (92.1)	166 009 (89.2)	183 762 (92.4)	178 116 (91.0)	<.001
Days therapeutic tablet given, n	7972	7449	8124	7962	.46

Days for which the status of supplement intake was unknown are excluded.

**Table VI:** Effects of 32-40 weeks of supplementation with daily preventive zinc supplements, daily micronutrient powder, or therapeutic zinc supplements for diarrhea on endline hemoglobin and anemia in rural Laotian children, stratified by baseline anemia status

Baseline anemia statuses	Preventive zinc	Micronutrient powder	Therapeutic zinc	Control	*P* value*
Anemic					
n	395	391	404	388	—
Hemoglobin, g/L	106.6 ± 0.5^a^	108.9 ± 0.5^b^	106.9 ± 0.5^a^	107.3 ± 0.5^a^	.005
Anemia at endline, %	59.3 ± 0.02^a^	50.1 ± 0.02^b^	57.1 ± 0.02^a^	56.1 ± 0.02^a^	.06
Not anemic					
n	341	295	321	332	
Hemoglobin, g/L	114.8 ± 0.5	115.3 ± 0.5	114.6 ± 0.5	115.1 ± 0.5	.77
Anemia at endline, %	26.3 ± 0.02	26.2 ± 0.03	24.1 ± 0.02	28.6 ± 0.02	.62

Values on the same row with different superscript are significantly different (*P* < .05) after adjustment for age, sex, and district baseline status.

* Values are mean (hemoglobin) or prevalence (anemia) ± SEM. Adjusted for age, sex, district, and respective baseline values; (%) marginal prevalence at endline.

**Table VII:** Complementary feeding practices among children participating in the Laos Zinc Study

*Indicators	Preventive zinc	Micronutrient powder	Therapeutic zinc	Control	*P* value
Minimum dietary diversity	29.0 (27.0-31.0)	30.2 (28.2-32.3)	28.6 (26.6-30.6)	29.4 (27.4-31.4)	.72
Animal source food	89.5 (88.2-90.7)	91.4 (90.2-92.6)	89.7 (88.4-90.9)	89.5 (88.2-90.6)	.18
Iron-rich foods	84.1 (82.7-85.6)	85.4 (84.0-86.9)	83.9 (82.5-85.3)	84.2 (82.8-85.6)	.44
Minimum meal frequency	60.8 (58.2-63.3)	61.0 (58.4-63.7)	60.2 (57.6-62.7)	62.4 (59.8-64.9)	.72

Complementary feeding practices assessed every 4 weeks throughout the study.

* Values represent the percentage of times children met the specified criteria as defined by the WHO^30^ and the 95% CIs around the point estimate.

**Table II:** Comparison of baseline characteristics between children who completed the study and those lost to follow-up

Characteristics	Completed the study	Lost to follow-up	*P* value
n (%)	2988 (87.1)	444 (12.9)	—
Age	14.2 ± 5.1	14.5 ± 5.0	.28
Length, cm	72.4 ± 5.6	72.7 ± 5.1	.30
Weight, kg	8.28 ± 1.31	8.41 ± 1.27	.06
MUAC, cm	13.8 ± 1.0	13.9 ± 1.1	.04
LAZ	−1.76 ± 1.08	−1.75 ± 0.95	.86
WAZ	−1.44 ± 1.01	−1.35 ± 0.98	.08
WLZ	−0.70 ± 0.96	−0.61 ± 1.00	.06
Stunting, n (%)	1188 (39.8)	166 (37.4)	.34
Underweight, n (%)	814 (27.2)	107 (24.1)	.16
Wasting, n (%)	241 (8.1)	35 (7.8)	.90
Hemoglobin, g/L	108 ± 11	107 ± 11	.18
Anemia, n (%)	1642 (54.9)	261 (58.8)	.13
Maternal BMI, kg/m^2^	21.4 ± 3.1	21.4 ± 3.1	.77
Maternal age, y	28.0 ± 6.0	27.5 ± 5.7	.09

Values represent mean ± SD for continuous variables and frequency (%) for dichotomous variables mean.

## Appendix

Supplement CONSORT 2010 checklist of information to include when reporting a randomised trial. Supplement
